# Insufficient Rest or Sleep and Its Relation to Cardiovascular Disease, Diabetes and Obesity in a National, Multiethnic Sample

**DOI:** 10.1371/journal.pone.0014189

**Published:** 2010-11-30

**Authors:** Anoop Shankar, Shirmila Syamala, Sita Kalidindi

**Affiliations:** 1 Department of Community Medicine, West Virginia University School of Medicine, Morgantown, West Virginia, United States of America; 2 Department of Medicine, West Virginia University School of Medicine, Morgantown, West Virginia, United States of America; 3 Department of Statistics, West Virginia University, Morgantown, West Virginia, United States of America; Louisiana State University, United States of America

## Abstract

**Background:**

A new question on insufficient rest/sleep was included in the 2008 Behavioral Risk Factor Surveillance System (BRFSS) for the 50 states, District of Columbia, and three US territories. No previous study, however, has examined perceived insufficient rest/sleep in relation to cardiovascular disease (CVD) or diabetes mellitus. We examined the association between self-reported insufficient rest/sleep and CVD, diabetes, and obesity in a contemporary sample of US adults.

**Methods:**

Multiethnic, nationally representative, cross-sectional survey (2008 BRFSS) participants were >20 years of age (n = 372, 144, 50% women). Self-reported insufficient rest/sleep in the previous month was categorized into four groups: zero, 1–13, 14–29, and 30 days. There were five outcomes: 1) any CVD, 2) coronary heart disease (CHD), 3) stroke, 4) diabetes mellitus, and 5) obesity (body mass index≥30 kg/m^2^). We employed multivariable logistic regression to calculate odds ratio (OR), (95% confidence interval (CI), of increasing categories of insufficient rest/sleep, taking zero days of insufficient rest/sleep as the referent category.

**Principal Findings:**

Insufficient rest/sleep was found to be associated with 1) any CVD, 2) CHD, 3) stroke, 4) diabetes mellitus, and 5) obesity, in separate analyses. Compared to those reporting zero days of insufficient sleep (referent), the OR (95% CI) associated with all 30 days of insufficient sleep was 1.67 (1.55–1.79) for any cardiovascular disease, 1.69(1.56–1.83) for CHD, 1.51(1.36–1.68) for stroke, 1.31(1.21–1.41) for diabetes, and 1.51 (1.43–1.59) for obesity.

**Conclusions:**

In a multiethnic sample of US adults, perceived insufficient rest/sleep was found to be independently associated with CHD, stroke, diabetes mellitus and obesity.

## Introduction

Sleep loss, long-term sleep deprivation, and perceived insufficient rest/sleep are common in modern society[1]. Nearly one-third of adults report sleeping <6 hours on average, leading some people to suggest that we live in a sleep-deprived society[2], [3]. Factors responsible for this change may include increases in environmental light, introduction of electric light, longer workdays/commuting time, shift and night work, expansion of manufacturing and service sectors to 24-hour-per-day operations, and the advent of television and internet[1], [4]. Especially in the current economic climate, Americans are more likely to be working longer hours or multiple jobs, and concerned about personal finance, employment and the economy—factors that are reported to be associated with perceived insufficient sleep in the 2009 Sleep in America Poll[2].

In response to an Institute of Medicine recommendation to expand surveillance of population sleep patterns[1], a new survey question on insufficient rest or sleep was included for the first time by the Centers for Disease Control and Prevention (CDC) in the 2008 Behavioral Risk Factor Surveillance System (BRFSS) core questionnaire for the 50 states, the District of Columbia, and the three US territories. A recent CDC report based on this survey found that an estimated 11.1% of Americans reported experiencing insufficient rest or sleep every day for the preceding 30 days and 30.7% of respondents reported no days of insufficient rest or sleep[5]. Perceived insufficient rest or sleep may be related to an underlying undiagnosed sleep disordered breathing[6], job strain[7], psychosocial stress[8], neighborhood effects[9] that may affect rest/sleep, depressive symptoms[10], certain endocrine disorders[11], or the effect of an unhealthy lifestyle[12], [13], all of which may predispose individuals to cardiovascular disease and diabetes mellitus. Even though previous reports have studied sleep duration, to our knowledge, no previous study has examined perceived insufficient rest or sleep in relation to cardiovascular disease and diabetes mellitus. Therefore, in this report, we examined the association between perceived insufficient rest or sleep and coronary heart disease, stroke, diabetes mellitus, and obesity after adjusting for main confounding factors in the 2008 BRFSS survey, which is a large, multiethnic sample of US adults.

## Methods

### Study population

The BRFSS is a federally funded nationally representative survey of the civilian, non-institutionalized, adult population aged 18 years or older. The survey is designed and conducted annually by the CDC in collaboration with the state health departments to monitor health-related behaviors and risk factors in the US population. The survey selects state-specific probability samples of households using a multistage cluster design to produce a nationally representative sample. The BRFSS uses random-digit dialing within blocks of telephone numbers to identify a probability sample of households with telephones in each state. In each household, one adult is randomly identified and interviewed. All 50 states, in addition to the District of Columbia, and the three US territories participated in the 2008 BRFSS. Detailed description of the BRFSS survey sample selection and study methodology are available online[14]. In 2008, the median cooperation rate was 75.0% and the median overall response rate was 53.3%[15].

To examine the association between insufficient rest/sleep and cardiovascular disease, diabetes mellitus, and obesity, out of the 414,509 BRFSS participants, we excluded subjects who were aged <20 years, pregnant, or who had missing information on variables included in the current analysis, including insufficient rest/sleep, physician diagnosed cardiovascular disease or diabetes, body mass index (BMI), smoking, education, or physical activity. This resulted in 372,144 adults (50% women) with complete covariate data who were included in the current analysis. Out of these, 42,644 subjects had any cardiovascular disease, 33,761 had coronary heart disease, 14,445 had stroke, 41,689 had diabetes mellitus, and 103,036 subjects were obese.

### Main outcomes of interest: Cardiovascular disease, diabetes mellitus, and obesity

The 2008 BRFSS core module asked questions about physician diagnosed history of coronary heart disease (descriptions used in the BRFSS questionnaire: “heart attack also called myocardial infarction”, “angina or coronary heart disease”), and stroke (description used in the BRFSS questionnaire: “stroke”). In the current study, we also defined “any cardiovascular disease” as the presence of self-reported coronary heart disease or stroke. Similarly, questions were asked on physician diagnosed history of diabetes mellitus (description used in the BRFSS questionnaire: “diabetes”). Body mass index (BMI) was calculated as self-reported weight in kilograms divided by height in meters squared. Obesity was defined as a BMI of ≥30 kg/m^2^ [16].

### Exposure measurements

The 2008 BRFSS survey included the question, “During the past 30 days, for about how many days have you felt you did not get enough rest or sleep?” Data from all sites were aggregated, and the numbers of days of perceived insufficient rest or sleep were categorized into four groups as zero days, 1–13 days, 14–29 days, and 30 days. This question was previously tested and validated in the 2006 BRFSS survey in four states (Delaware, Hawaii, New York, and Rhode Island)[17]. Also, national estimates of insufficient rest or sleep according to these categories have been recently published by the CDC[5].

Age, gender, race/ethnicity, smoking status, alcohol intake, level of education, physical activity were assessed using a standardized questionnaire. Individuals who had not smoked ≥100 cigarettes in their lifetimes were classified as never smokers; those who had smoked ≥100 cigarettes in their lifetimes were classified as former smokers or current smokers based on their response to the question on current smoking. Heavy alcohol drinking was defined as men who reported having more than 2 drinks per day, or women that reported having more than 1 drink per day. Education was categorized into below high school, high school, or above high school education. Employment status was categorized as employed, unemployed, retired, or unable to work. BMI was categorized into <25, 25–29, ≥30 kg/m^2^. Subjects were classified as having no regular exercise if they reported not to be participating in any physical activities such as running, calisthenics, golf, gardening, or walking for exercise during the previous month.

### Statistical analysis

We examined the characteristics of the study sample by calculating the mean values of continuous variables and frequencies of categorical variables. Perceived insufficient rest or sleep were categorized into four groups as zero days, 1–13 days, 14–29 days, and 30 days. We had five outcomes of interest: 1) any cardiovascular disease, 2) coronary heart disease, 3) stroke, 4) diabetes mellitus, and 5) obesity. We used logistic regression models to calculate odds ratio [(OR) (95% confidence interval (CI)] of each outcome of interest associated with increasing categories of insufficient rest/sleep, taking zero days of insufficient rest/sleep as the referent category. We used two nested logistic regression models: the age and sex-adjusted model and the multivariable model, additionally adjusting for race-ethnicity (non-Hispanic whites, non-Hispanic blacks, Mexican Americans, others), education categories (<high school, high school, >high school), smoking (never, former, current), employment status (employed, unemployed, retired, unable to work), heavy drinker (no, yes), BMI categories (<25, 25–29, ≥30 kg/m^2^), no regular exercise (yes, no). Trends in the OR of each outcome across increasing insufficient rest/sleep category were determined by modeling these categories as an ordinal variable. Appropriate BRFSS survey weights that account for unequal probabilities of selection, oversampling, and non-response were applied for all analyses using SUDAAN (version 8.0; Research Triangle Institute, Research Triangle Park, NC) and SAS (version 9.2; SAS Institute, Cary, NC) software; SEs were estimated using the Taylor series linearization method.

## Results

**Figure 1:**
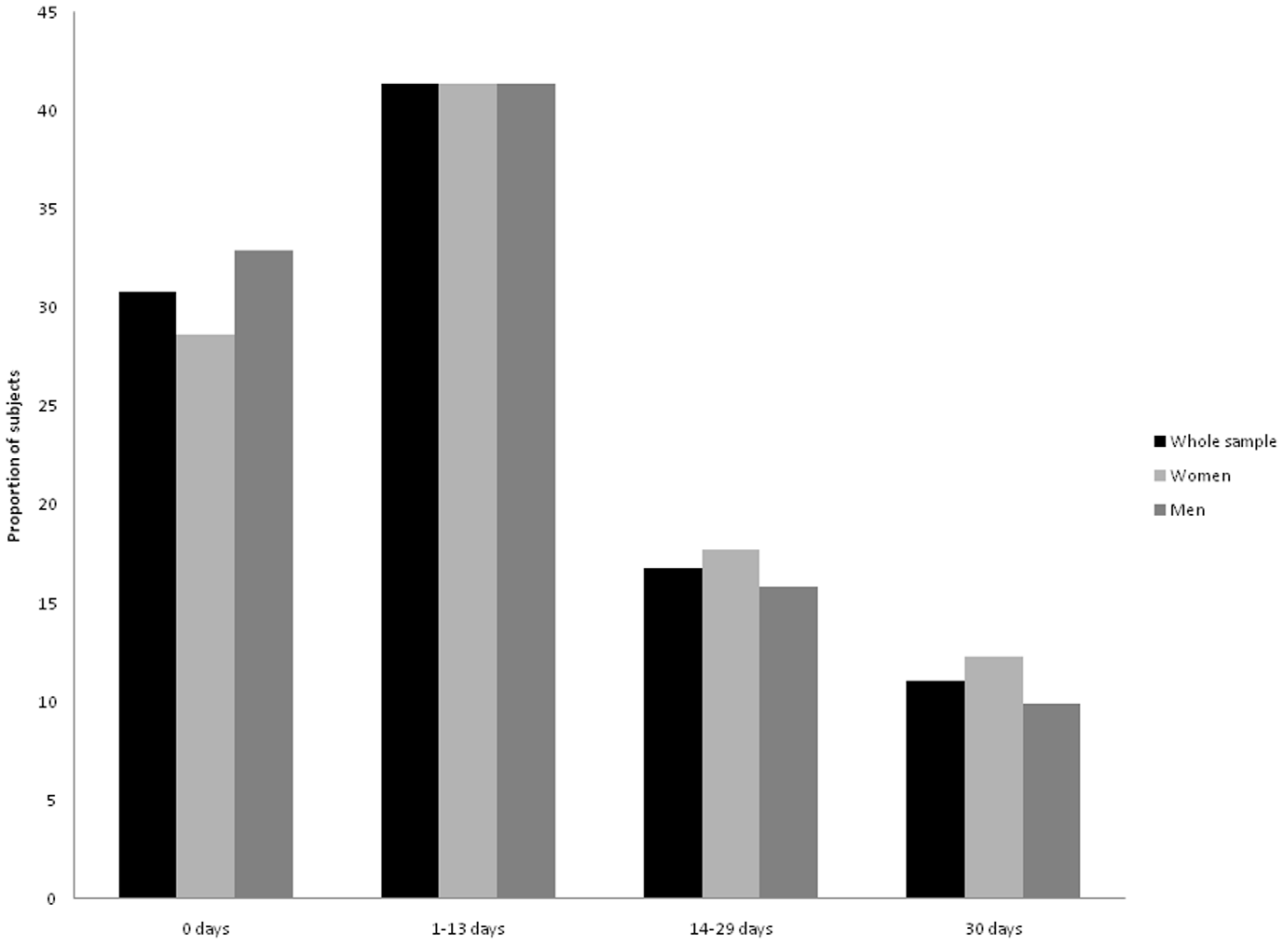
Insufficient rest or sleep among US adults. X-axis: Categories of insufficient rest or sleep in the past month. Y-axis: Weighted proportion (%) of subjects within each insufficient rest/sleep category.

Table 1 presents the characteristics of the study population. Half of the study sample were women, approximately 17% were subjects ≥65 years of age, 10% were blacks, while 14% were Mexican Americans. Approximately 62% of study subjects had above high school education, 69% were employed, 19% were current smokers, 5% were heavy alcohol drinkers and 25% reported no regular exercise. Furthermore, approximately 27% of subjects were obese, 9% had diabetes mellitus, and 8% had any cardiovascular disease, including coronary heart disease or stroke. Figure 1 presents the proportion of adults within each category on insufficient rest/sleep. Overall, 31% subjects reported zero days of insufficient rest/sleep while 11% reported all 30 days of insufficient rest/sleep in the past month. Women were more likely than men to report all 30 days of insufficient rest/sleep (12% vs. 10%).

**Table 1:** Baseline characteristics of the study population (N = 372,144).

Characteristics	Percentages or mean values ± standard error (SE)
Women, %	50.0±0.2
Age groups, %	
<25 years	8.5±0.2
25 to 28 years	18.8±0.2
35 to 44 years	20.1±0.1
45 to 64 years	35.3±0.2
≥65 years	17.3±0.1
Race-ethnicity, %	
Non-Hispanic whites	69.3±0.2
Non-Hispanic blacks	9.7±0.1
Mexican Americans	14.2±1.2
Others	6.7±0.1
Education categories, %	
Below high school	10.2±0.1
High school	27.5±0.2
Above high school	62.3±0.2
Employment status, %	
Employed	69.4±0.2
Unemployed	6.4±0.1
Retired	18.4±0.1
Unable to work	5.8±0.1
Smoking, %	
Never smoker	55.8±0.2
Former smoker	25.5±0.1
Current smoker	18.7±0.1
Heavy alcohol drinker, %	5.3±0.1
No regular exercise, %	25.1±0.2
Body mass index categories, %	
Normal weight	35.7±0.2
Over weight	37.0±0.2
Obese	27.3±0.2
Diabetes mellitus, %	8.9±0.09
Cardiovascular disease, %	8.3±0.08

Table 2 presents the association between increasing categories of insufficient rest/sleep and any cardiovascular disease. Overall, there was a positive association between insufficient rest/sleep and any cardiovascular disease both in the age, sex-adjusted model as well as the multivariable-adjusted model. Models evaluating trend in this association were also statistically significant (p-trend<0.0001). Furthermore, the positive association between insufficient rest/sleep and any cardiovascular disease was consistently present in separate analysis among men (p-trend<0.0001) and women (p-trend<0.0001).

**Table 2:** Association between insufficient rest/sleep and any cardiovascular disease (CVD).

Categories of insufficient rest/sleep	No. at risk	No. with CVD	Age, sex-adjusted odds ratio (95% confidence intervals)	Multivariable odds ratio (95% confidence intervals)*
Whole cohort				
0 days	133265	18561	1 (referent)	1 (referent)
1–13 days	144187	12014	0.93 (0.88–0.98)	1.02 (0.97–1.08)
14–29 days	55983	5920	1.46 (1.37–1.56)	1.33 (1.24–1.42)
30 days	38709	6149	2.25 (2.10–2.40)	1.67 (1.55–1.79)
p-trend			<0.0001	<0.0001
Men+				
0 days	56644	9652	1 (referent)	1 (referent)
1–13 days	54551	5412	0.90 (0.84–0.97)	1.03 (0.95–1.11)
14–29 days	19665	2399	1.39 (1.26–1.53)	1.31 (1.18–1.45)
30 days	13711	2578	2.03 (1.83–2.26)	1.56 (1.39–1.75)
p-trend			<0.0001	<0.0001
Women+				
0 days	76621	8909	1 (referent)	1 (referent)
1–13 days	89636	6602	0.97 (0.91–1.04)	1.04 (0.97–1.11)
14–29 days	36318	3521	1.57 (1.44–1.71)	1.37 (1.26–1.50)
30 days	24998	3571	2.50 (2.30–2.72)	1.79 (1.63–1.96)
p-trend			<0.0001	<0.0001

*Adjusted for age (years), sex (men, women), race-ethnicity (non-Hispanic whites, non-Hispanic blacks, Mexican Americans, others), education categories (<high school, high school, >high school), smoking (never, former, current), employment status (employed, unemployed, retired, unable to work), heavy drinker (no, yes), body mass index categories (<25, 25–29, ≥30 kg/m^2^), no regular exercise (yes, no).

+p-interaction by gender = 0.312.

Tables 3 and 4 present the association between increasing categories of insufficient rest/sleep and components of any cardiovascular disease, including coronary heart disease and stroke, respectively. The positive association between insufficient rest/sleep and cardiovascular disease was consistently present in separate analysis examining coronary heart disease (Table 3) and stroke (Table 4) as the outcome of interest. Also, the positive association was consistently present in subgroup analysis by gender.

**Table 3 pone-0014189-t003:** Association between insufficient rest/sleep and coronary heart disease (CHD).

Categories of insufficient rest/sleep	No. at risk	No. with CHD	Age, sex-adjusted odds ratio (95% confidence intervals)	Multivariable odds ratio (95% confidence intervals)*
Whole cohort				
0 days	133265	14553	1 (referent)	1 (referent)
1–13 days	144187	9516	0.97(0.92–1.03)	1.07(1.01–1.13)
14–29 days	55983	4793	1.54(1.43–1.65)	1.41(1.31–1.52)
30 days	38709	4899	2.23(2.07–2.40)	1.69(1.56–1.83)
p-trend			<0.0001	<0.0001
Men+				
0 days	56644	8168	1 (referent)	1 (referent)
1–13 days	54551	4655	0.94(0.87–1.02)	1.06(0.98–1.15)
14–29 days	19665	2044	1.40(1.26–1.56)	1.33(1.20–1.49)
30 days	13711	2172	1.98(1.77–2.21)	1.55(1.37–1.74)
p-trend			<0.0001	<0.0001
Women+				
0 days	76621	6385	1 (referent)	1 (referent)
1–13 days	89636	4861	1.03(0.95–1.11)	1.09(1.01–1.18)
14–29 days	36318	2749	1.74(1.59–1.92)	1.53(1.39–1.69)
30 days	24998	2727	2.59(2.35–2.84)	1.87(1.69–2.07)
p-trend			<0.0001	<0.0001

*Adjusted for age (years), sex (men, women), race-ethnicity (non-Hispanic whites, non-Hispanic blacks, Mexican Americans, others), education categories (<high school, high school, >high school), smoking (never, former, current), employment status (employed, unemployed, retired, unable to work), heavy drinker (no, yes), body mass index categories (<25, 25–29, ≥30 kg/m^2^), no regular exercise (yes, no).

+p-interaction by gender = 0.192.

**Table 4 pone-0014189-t004:** Association between insufficient rest/sleep and stroke.

Categories of insufficient rest/sleep	No. at risk	No. with stroke	Age, sex-adjusted odds ratio (95% confidence intervals)	Multivariable odds ratio* (95% confidence intervals)
Whole cohort				
0 days	133265	6300	1 (referent)	1 (referent)
1–13 days	144187	3903	0.87(0.80–0.94)	0.96(0.89–1.05)
14–29 days	55983	1988	1.31(1.19–1.44)	1.15(1.04–1.27)
30 days	38709	2254	2.18(1.98–2.40)	1.51(1.36–1.68)
p-trend			<0.0001	<0.0001
Men+				
0 days	56644	2680	1 (referent)	1 (referent)
1–13 days	54551	1342	0.80(0.70–0.92)	0.94(0.82–1.09)
14–29 days	19665	694	1.29(1.10–1.51)	1.18(1.01–1.39)
30 days	13711	828	2.16(1.85–2.52)	1.50(1.27–1.78)
p-trend			<0.0001	<0.0001
Women+				
0 days	76621	3620	1 (referent)	1 (referent)
1–13 days	89636	2561	0.92(0.84–1.02)	0.99(0.90–1.09)
14–29 days	36318	1294	1.33(1.18–1.50)	1.14(1.01–1.29)
30 days	24998	1426	2.21(1.96–2.49)	1.53(1.35–1.75)
p-trend			<0.0001	<0.0001

*Adjusted for age (years), sex (men, women), race-ethnicity (non-Hispanic whites, non-Hispanic blacks, Mexican Americans, others), education categories (<high school, high school, >high school), smoking (never, former, current), employment status (employed, unemployed, retired, unable to work), heavy drinker (no, yes), body mass index categories (<25, 25–29, ≥30 kg/m^2^), no regular exercise (yes, no).

+p-interaction by gender = 0.573.

Table 5 presents the association between increasing categories of insufficient rest/sleep and diabetes mellitus. There was a positive association between insufficient rest/sleep and diabetes mellitus both in the age, sex-adjusted model as well as the multivariable-adjusted model. Models evaluating trend in this association were also statistically significant (p-trend<0.0001). Also, the positive association between insufficient rest/sleep and diabetes mellitus was consistently present in separate analysis among men (p-trend<0.0001) and women (p-trend<0.0001).

**Table 5 pone-0014189-t005:** Association between insufficient rest/sleep and diabetes mellitus.

Categories of insufficient rest/sleep	No. at risk	No. with diabetes	Age, sex-adjusted odds ratio (95% confidence intervals)	Multivariable odds ratio (95% confidence intervals)*
Whole cohort				
0 days	133265	16993	1 (referent)	1 (referent)
1–13 days	144187	12964	0.95(0.9–0.99)	1.03(0.97–1.08)
14–29 days	55983	6058	1.26(1.18–1.34)	1.15(1.07–1.23)
30 days	38709	5674	1.75(1.63–1.87)	1.31(1.21–1.41)
p-trend			<0.0001	<0.0001
Men+				
0 days	56644	8055	1 (referent)	1 (referent)
1–13 days	54551	5218	0.95(0.87–1.02)	1.06(0.98–1.15)
14–29 days	19665	2148	1.17(1.06–1.30)	1.12(1.01–1.24)
30 days	13711	2116	1.56(1.40–1.74)	1.23(1.09–1.39)
p-trend			<0.0001	<0.0001
Women+				
0 days	76621	8938	1 (referent)	1 (referent)
1–13 days	89636	7746	0.96(0.90–1.02)	1.01(0.94–1.08)
14–29 days	36318	3910	1.36(1.25–1.47)	1.19(1.09–1.30)
30 days	24998	3558	1.94(1.79–2.11)	1.38(1.26–1.52)
p-trend			<0.0001	<0.0001

*Adjusted for age (years), sex (men, women), race-ethnicity (non-Hispanic whites, non-Hispanic blacks, Mexican Americans, others), education categories (<high school, high school, >high school), smoking (never, former, current), employment status (employed, unemployed, retired, unable to work), heavy drinker (no, yes), body mass index categories (<25, 25–29, ≥30 kg/m^2^), no regular exercise (yes, no).

+p-interaction by gender = 0.244.

Finally, Table 6 presents the association between increasing categories of insufficient rest/sleep and obesity. There was a positive association between insufficient rest/sleep and obesity both in the age, sex-adjusted model as well as the multivariable-adjusted model. Models evaluating trend in this association were also statistically significant (p-trend<0.0001). The positive association persisted in separate analysis among men (p-trend<0.0001) and women (p-trend<0.0001).

**Table 6:** Association between insufficient rest/sleep and obesity.

Categories of insufficient rest/sleep	No. at risk	No. with obesity	Age, sex-adjusted odds ratio (95% confidence intervals)	Multivariable odds ratio (95% confidence intervals)*
Whole cohort				
0 days	133265	32929	1 (referent)	1 (referent)
1–13 days	144187	38493	1.05 (1.01–1.09)	1.10 (1.06–1.14)
14–29 days	55983	17900	1.34 (1.28–1.41)	1.36 (1.30–1.43)
30 days	38709	13714	1.62 (1.54–1.70)	1.51 (1.43–1.59)
p-trend			<0.0001	<0.0001
Men+				
0 days	56644	14628	1 (referent)	1 (referent)
1–13 days	54551	15012	1.04 (0.98–1.11)	1.08 (1.02–1.14)
14–29 days	19665	6311	1.30 (1.20–1.40)	1.31 (1.21–1.41)
30 days	13711	4960	1.58 (1.45–1.71)	1.52 (1.40–1.65)
p-trend			<0.0001	<0.0001
Women+				
0 days	76621	18301	1 (referent)	1 (referent)
1–13 days	89636	23481	1.04 (1.00–1.09)	1.11 (1.06–1.17)
14–29 days	36318	11589	1.39 (1.31–1.47)	1.41 (1.33–1.50)
30 days	24998	8754	1.66 (1.56–1.77)	1.49 (1.40–1.59)
p-trend			<0.0001	<0.0001

*Adjusted for age (years), sex (men, women), race-ethnicity (non-Hispanic whites, non-Hispanic blacks, Mexican Americans, others), education categories (<high school, high school, >high school), smoking (never, former, current), employment status (employed, unemployed, retired, unable to work), heavy drinker (no, yes), no regular exercise (yes, no).

+p-interaction by gender = 0.375.

In a supplementary analysis, to examine the robustness of our findings, we examined the association between insufficient rest/sleep and CVD employing an alternate, finer grouping of insufficient rest/sleep (zero days, 1–7 days, 8–14 days, 15–29 days, and 30 days); the results were found to be essentially similar. Compared to zero days of insufficient rest/sleep (referent), the multivariable OR (95% CI) of CVD was 1.14 (0.98–1.34) for 1–7 days, 1.22 (1.11–1.34) for 8–14 days, 1.33 (1.24–1.43) for 15–29 days, and 1.65 (1.51–1.80) for 30 days; p-trend<0.0001. In a second supplementary analysis, we calculated the population attributable risk of each outcome examined in Tables 2 to [Table pone-0014189-t003] [Table pone-0014189-t004] [Table pone-0014189-t005] 6. Approximately 11% of US adults reported insufficient rest/sleep on all 30 days. The population attributable risk associated with insufficient rest/sleep for all 30 days was 6.9% for any CVD, 7.1% for CHD, 5.3% for stroke, 3.3% for diabetes mellitus, and 5.3% for obesity.

## Discussion

In a contemporary, nationally representative sample of US adults, increasing categories of insufficient rest or sleep was found to be positively associated with 1) any cardiovascular disease, 2) coronary heart disease, 3) stroke, 4) diabetes mellitus and 5) obesity, in separate analyses. The association was found to be independent of age, gender, race-ethnicity, education, smoking, employment, heavy alcohol drinking, and lack of regular exercise and persisted in separate analysis among men and women. Therefore, the single question on perceived lack of rest or sleep, introduced nationally by CDC for the first time in the 2008 BRFSS in all 50 states, the District of Columbia, and the three US territories as a tool for studying sleep patterns, also appears to be a good predictor of cardiovascular disease and diabetes in relation to sleep loss.

In the current study, the magnitude of the observed association between insufficient sleep and various outcomes, the persistence of this association after multivariable adjustment of confounders, and the consistency of these findings in subgroup analysis by gender suggest that these findings are less likely to be due to chance. Previous studies have found that self-reported short sleep duration is associated with cardiovascular disease[18]–[21], diabetes mellitus[22]–[24] and obesity[25]–[27]. Some studies have reported that long sleep duration is also associated with cardiovascular disease[18], [19], [21]. Our findings on the association between perceived insufficient rest or sleep are consistent with these previous reports. While short sleep duration may be a contributing factor for perceived insufficient rest or sleep, long sleep duration may be a manifestation of it. Furthermore, perceived insufficient rest or sleep may be related to factors which may predispose individuals to increased likelihood of cardiovascular disease and diabetes mellitus, such as an underlying sleep apnea or sleep disordered breathing[6], job strain[7], psychosocial stress[8], neighborhood effects[9], depressive symptoms[10], endocrine disorders[11], or the effect of lifestyle choices[12], [13], including late-night television watching or internet use, consumption of stimulants, cigarette smoking, heavy alcohol consumption, and physical inactivity.

Other contributing factors of perceived rest or sleep loss that needs further investigation as cardiovascular disease risk factors include occupational factors such as extended work schedules, jet lag, or shift work, resulting in irregular sleep schedules[1]. Additionally, other sleep disorders such as insomnia, restless legs syndrome, narcolepsy, and circadian rhythm disorders, can cause sleep loss[1].

The advantages of our study include the following: the nationally representative nature with study samples from all 50 US states, the District of Columbia, and the three territories; inclusion of both men and women, and all race-ethnicities including minorities; and the large sample size. The findings in this report are subject to several limitations. First, the BRFSS was a cross-sectional study. Therefore, the temporal nature of the association between perceived insufficient rest or sleep and cardiovascular disease and diabetes mellitus cannot be established from our study. The definitions of “insufficient” sleep and “rest” and responses to the BRFSS survey question were subjective. Therefore, this analysis cannot be compared directly with objective studies of sleep duration or polysomnographic measures of sleep disordered breathing. Second, the outcome definitions of cardiovascular disease, diabetes, and obesity were based on self-reported information which could have resulted in misclassification. We believe this misclassification is likely to be nondifferential and therefore likely to underestimate the true associations. Third, information on shift work was not available in the study. Therefore it is possible that the observed association between insufficient rest/sleep and CVD is due to the confounding effect of shift work. Fourth, the definitions of “insufficient” sleep and “rest” and responses to the BRFSS survey question were subjective. As a measure of lack of sleep, this question may introduce exposure misclassification and bias our results. Therefore, this analysis may not be compared directly with objective studies of sleep duration or polysomnographic measures of sleep disordered breathing. Fifth, despite statistically significant associations, the population attributable risk of insufficient rest/sleep on various outcomes considered in the study was modest and ranged from 3.3% to 7.1%, suggesting only a small fraction of these outcomes are explained by insufficient rest/sleep at the population level. Finally, although similar questions on perceived rest/sleep have been included in widely tested and validated sleep quality instruments such as the Pittsburgh Sleep Quality Index and the Epworth Sleepiness Scale, to our knowledge, the specific question on insufficient rest/sleep used in the BRFSS survey has never been tested. It is possible that the reliability and validity of the question used in the current study is low and therefore our results may be biased by exposure misclassification. Future studies examining the association between the insufficient rest/sleep question used in BRFSS and objectively measured sleep duration (e.g. actigraphy) are needed.

In summary, in a multiethnic sample of US adults, increasing categories of insufficient rest or sleep was found to be positively associated with coronary heart disease, stroke, diabetes mellitus and obesity. The association was found to be independent of age, gender, race-ethnicity, education, smoking, employment, heavy alcohol drinking, and lack of regular exercise and persisted in separate analysis among men and women. The single question on perceived lack of rest or sleep, introduced nationally by CDC for the first time in the 2008 BRFSS appears to be a good predictor of cardiovascular disease, diabetes and obesity.
